# 补肾活血通络方代谢产物对多发性骨髓瘤KM3细胞增殖的影响及其作用机制

**DOI:** 10.3760/cma.j.cn121090-20241209-00547

**Published:** 2025-07

**Authors:** 镜铂 史, 昌年 李, 文健 魏, 继元 丁, 国栋 马, 璐璐 李, 亚茹 王, 亦桐 路, 杰 徐, 伟 郑, 琰 王, 敬毅 王, 瑞荣 徐, 思远 崔

**Affiliations:** 1 山东中医药大学第一临床医学院，济南 250014 The First School of Clinical Medicine, Shandong University of Traditional Chinese Medicine, Jinan 250014, China; 2 山东中医药大学附属医院血液病科，济南 250014 Department of Hematology, Affiliated Hospital of Shandong University of Traditional Chinese Medicine, Jinan 250014, China

**Keywords:** 多发性骨髓瘤, 线粒体动力学, 自噬, 网络药理学, Multiple myeloma, Mitochondrial dynamics, Autophagy, Network pharmacology

## Abstract

**目的:**

探索补肾活血通络方代谢产物对多发性骨髓瘤（MM）KM3细胞增殖的影响及其作用机制。

**方法:**

分别用含3％、6％、9％、12％的补肾活血通络方代谢产物处理对数生长期的MM KM3细胞株，CCK-8法检测KM3细胞活力，采用流式细胞术和TUNEL染色法检测细胞凋亡和坏死情况。通过透射电子显微镜观察细胞结构，荧光定量PCR和Western blot检测动力相关蛋白1（Drp1）、线粒体裂变蛋白1（Fis1）、线粒体分裂因子（MFF）、PTEN诱导激酶1（Pink1）和E3泛素连接酶（Parkin）的mRNA和蛋白表达水平。通过高效液相色谱-串联质谱（HPLC-MS/MS）技术结合网络药理学对补肾活血通络方治疗MM的药效基础与作用靶点进行反向验证。

**结果:**

补肾活血通络方代谢产物抑制KM3细胞增殖，诱导细胞凋亡，并呈剂量依赖性。透射电镜观察细胞结构显示，与对照组相比，3％、6％、9％、12％代谢产物组细胞内线粒体分裂和细胞自噬数量增多，并随代谢产物浓度的升高而递增。Drp1、Fis1、MFF、Pink1、Parkin的mRNA和蛋白的表达水平较对照组均升高（*P*值均<0.05），其中12％代谢产物组与对照组的差异最为显著（*P*<0.01）。通过HPLC-MS/MS技术从补肾活血通络方中鉴别筛选出121个活性成分，与MM有474个交集靶点，富集分析结果表明，补肾活血通络方可能通过芹菜素、黄藤素等主要活性成分调控肿瘤坏死因子、核因子κB、线粒体自噬等通路发挥抗肿瘤作用。

**结论:**

补肾活血通络方可抑制MM KM3细胞增殖，其机制可能与调控线粒体分裂、诱导细胞自噬相关。

## Abstract (en)

**Objective:**

To evaluate the effects and underlying mechanisms of metabolites derived from the kidney-reinforcing, blood circulation-activating, and collateral dredging decoction on the proliferation of multiple myeloma（MM）KM3 cells.

**Methods:**

MM KM3 cells in the logarithmic growth phase were treated with 3％, 6％, 9％, or 12％ metabolites of kidney-reinforcing, blood circulation-activating, and collateral dredging decoction. Cell viability was assessed using the CCK-8 assay. Apoptosis and necrosis were evaluated using flow cytometry and TUNEL staining. Mitochondrial and cellular ultrastructural changes were examined using transmission electron microscopy. mRNA and protein expression levels of dynamin-related protein 1（Drp1）, mitochondrial fission protein 1（Fis1）, mitochondrial fission factor（MFF）, PTEN-induced kinase 1（Pink1）, and E3 ubiquitin ligase（Parkin）were determined through quantitative real-time PCR and western blotting. High-performance liquid chromatography-tandem mass spectrometry（HPLC-MS/MS）combined with network pharmacology, was utilized for reverse verification of the pharmacodynamic mechanisms and therapeutic targets underlying the anti-MM activity of this decoction.

**Results:**

The metabolites of the kidney-reinforcing, blood circulation-activating, and collateral dredging decoction inhibited KM3 cell proliferation and induced apoptosis in a dose-dependent manner. Transmission electron microscopy revealed increased mitochondrial fission and autophagic structures, with effects intensifying at higher metabolite concentrations. mRNA and protein expression of Drp1, Fis1, MFF, Pink1, and Parkin were significantly upregulated in treatment groups compared to controls（*P*<0.05）, with the most pronounced effects observed in the 12％ metabolite group（*P*<0.01）. HPLC-MS/MS identified 121 bioactive compounds in BHTF, which shared 474 overlapping targets with MM. Enrichment analysis suggested that BHTF exerts antitumor effects primarily through apigenin, palmatine, and other key components by modulating TNF, NF-κB, and mitophagy pathways.

**Conclusion:**

The kidney-reinforcing and blood circulation-activating and collateral dredging decoction suppresses the proliferation of MM KM3 cells, potentially through mechanisms involving the regulation of mitochondrial dynamics and induction of autophagy.

多发性骨髓瘤（MM）是一种以骨髓中单克隆浆细胞异常增殖为特征的血液系统恶性肿瘤[1]，随着蛋白酶体抑制剂、免疫调节药物、嵌合抗原受体T细胞免疫治疗等疗法的应用，MM患者的预后得到了改善，但仍无法治愈[2]。线粒体动力学涵盖了线粒体分裂及自噬等过程，维持线粒体网络动态平衡并满足细胞能量代谢[3]。研究显示，线粒体的融合分裂与细胞周期、凋亡、自噬等关系密切[4]，MM的发生发展也与线粒体动力学联系紧密[5]–[6]。线粒体分裂过程主要受动力相关蛋白1（Drp1）、线粒体裂变蛋白1（Fis1）和线粒体分裂因子（MFF）调控[7]。缺氧可诱导线粒体向核周聚集，某些应激条件下通过微管定向运输至溶酶体，引发线粒体自噬，线粒体自噬主要由PTEN诱导激酶1（Pink1）/E3泛素连接酶（Parkin）介导[8]。

MM属于中医学中“骨痹”等范畴，近代医家认为其病因分内外两种，内因肝肾失调，瘀毒内生，久则肾精不足，无以生髓化血，并见虚劳诸证；复因外感邪气，深入骨髓，毒瘀阻滞[9]。外感内伤相合则肾虚血瘀，经络不通，治宜补肾活血通络[10]。补肾活血通络方为山东中医药大学附属医院经验方，在MM前期治疗中的治疗效果良好。本研究基于线粒体动力学探讨补肾活血通络方治疗MM的可能机制，并通过高效液相色谱-串联质谱（HPLC-MS/MS）技术和网络药理学进行验证。

## 材料与方法

1. 细胞与动物：人MM KM3细胞株由山东大学齐鲁医院肿瘤中心王鲁群教授惠赠；SPF级雄性SD大鼠，6周龄，体重230～250 g，购于北京维通利华实验动物技术有限公司，生产许可证号为：SCXK（京）2021-0006，大鼠饲养于山东中医药大学附属医院（山东省中医院）动物实验中心层流架内（符合SPF标准），温度20～25 °C，湿度55％～60％。鼠笼、饮用水、饲料、垫料均经高压灭菌处理。适应性饲养7 d后进行实验。实验方案经山东中医药大学附属医院实验动物伦理委员会审批（批准号：SDSZYYAWE20240607001）。

2. 药品、试剂与仪器：补肾活血通络方（杜仲15 g，桑寄生15 g，续断15 g，山茱萸15 g，党参15 g，茯苓15 g，当归15 g，赤芍15 g，延胡索15 g，牛膝10 g，没药6 g，全蝎6 g，蜈蚣2条，细辛3 g，三七10 g，莪术10 g，补骨脂30 g，黄精10 g）饮片由山东中医药大学附属医院中药制剂实验室（国家三级中药制剂实验室）配制成中药药液，灌装于250 ml输液瓶中，流通蒸汽于105 °C灭菌，无菌试验阴性后加青霉素100 U/ml，链霉素0.1 mg/ml，正压过滤除菌，4 °C保存备用，应用时平衡至室温；RPMI-1640培养基、青霉素/链霉素溶液、BCA蛋白定量浓度测定试剂盒、Annexin V-FITC/PI细胞凋亡检测试剂盒均购自大连美仑生物技术有限公司，FBS购自苏州依科赛生物科技有限公司，CCK-8细胞增殖毒性检测试剂盒、总RNA提取试剂盒和反转录试剂盒均购自广州信天翁生物技术有限公司，PCR引物购自北京擎科生物技术有限公司；PVDF膜购自美国Millipore公司；Western blot内参、一抗、二抗均购自武汉三鹰生物技术有限公司；乙腈、甲醇，为质谱纯，购自德国Merck KGaA公司；超高效液相色谱仪、高分辨质谱仪购自美国Thermo公司；Light Cycler480Ⅱ实时荧光定量PCR仪购自瑞士Roche公司；流式细胞仪购自美国Agilent公司。

3. 补肾活血通络方代谢产物的制备：补肾活血通络方药材加水煎煮后过滤浓缩为浸膏，含生药2 g/ml。按照换算剂量，实验组按体重19.6 g/kg予生药灌胃，每日1次，连续7 d。采血前1 d禁食，末次给药后2 h无菌条件下于腹主动脉取血，分离血清，用微孔滤膜过滤除菌后分别与RPMI1640培养基和FBS配制为3％（3％代谢产物+12％ FBS）、6％（6％代谢产物+9％ FBS）、9％（9％代谢产物+6％ FBS）、12％（12％代谢产物+3％ FBS）的补肾活血通络方代谢产物备用，设置含15％FBS的RPMI-1640培养基为对照组。

4. 细胞培养：人多发性骨髓瘤KM3细胞，用含15％ FBS的RPMI-1640培养基培养，置于37 °C、5％ CO_2_、饱和湿度培养箱中进行常规传代培养，1～2 d换液、传代1次，倒置显微镜观察细胞生长状态，取对数生长期细胞进行实验。

5. CCK-8法检测细胞活力：取对数生长期KM3细胞在RPMI-1640培养基中培养，调整细胞浓度为1×10^6^/ml，接种于96孔板中，每孔100 µl。设置1个对照组、4个实验组和1个空白组，对照组加入FBS，实验组分别加入含3％、6％、9％、12％的补肾活血通络方代谢产物培养，另设5个无细胞孔加入等体积培养基作为空白组。每孔最终体积为200 µl，每个浓度设置5个复孔，于37 °C、5％ CO_2_环境下分别培养12、24、48和72 h后，每孔加入10 µl CCK-8试剂继续培养2 h，酶标仪读取450 nm处各孔吸光度（*A*）值。细胞存活率=（*A*_实验组_−*A*_空白组_）/（*A*_对照组_−*A*_空白组_）×100％，增殖抑制率=1−细胞存活率。

6. Annexin V-FITC/PI双染法流式细胞术检测细胞凋亡：用不同浓度代谢产物培养KM3细胞72 h，放入离心机1 000×*g*离心5 min后收集细胞，用磷酸盐缓冲液（PBS）重悬细胞并计数，每组样本控制细胞数为1×10^5^个，1 000×*g*离心5 min后弃上清，加入195 µl的Annexin V-FITC结合液重悬细胞，加入5 µl Annexin-V-FITC和10 µl碘化丙啶染色液混匀，室温、避光孵育15 min后置于冰浴中，使用流式细胞仪检测细胞凋亡情况并计算细胞凋亡率。

7. TUNEL染色法检测细胞凋亡：用不同浓度代谢产物干预细胞72 h后，收集各组细胞加入染色剂后室温孵育30 min，荧光显微镜下计算阳性细胞数，红色阳性细胞为凋亡细胞。凋亡率（％）=（阳性细胞数/细胞总数）×100％。

8. 透射电镜观察KM3细胞结构变化：弃去培养液加入电镜固定液4 °C固定4 h，细胞低速离心，1％琼脂糖包裹，0.1 mol/L PBS漂洗3次，每次15 min。洗涤后加入1％的锇酸，2 h后弃置液体，0.1 mol/L PBS漂洗3次，每次15 min。细胞组织依次浸入不同浓度乙醇中进行脱水，每次15 min，而后在100％丙酮中浸入15 min。渗透、包埋后用超薄切片机切成60～80 nm厚度的超薄切片。铀铅双染色（2％醋酸铀饱和酒精溶液和枸橼酸铅各染色15 min），切片室温干燥过夜。透射电子显微镜下观察，采集图像分析。

9. 荧光定量PCR检测Drp1、Fis1、MFF、Pink1、Parkin mRNA表达水平：收集细胞，冰上裂解，TRIzol法提取细胞总RNA，使用酶标仪测定浓度。按照逆转录试剂盒中说明书步骤逆转录提取cDNA后，qPCR检测mRNA的表达，根据PCR试剂盒说明书配置反应总体系（10 µl）：1 µl cDNA、5 µl PCR试剂、0.2 µl上游引物、0.2 µl下游引物和3.6 µl无酶水，引物序列见表1。反应条件：95 °C预变性30 s，95 °C 10 s，60 °C 30 s，40个循环，以GAPDH为内参基因，采用2^−ΔΔCt^法计算各个基因的相对表达量。

**表1:** PCR引物信息

引物名称	引物序列（5′→3′）
Drp1-F	GCGGTGGTGCTAGAATTTGTTAT
Drp1-R	CCGCTTCACCAGTAACTCAAATG
Fis1-F	CTCATTCAGGACTGGCCGTT
Fis1-R	TTCCCGACTGCTCATCAACC
MFF-F	CACCACCTCGTGTACTTACGC
MFF-R	CCGCTCTCTTTTTAGTCTGCC
Pink1-F	GGGAGTATGGAGCAGTCACTTAC
Pink1-R	GCAGGGTACAGGGATAGTTCTTC
Parkin-F	GGCTGTCCCAACTCCTTGATTAA
Parkin-R	GCTTCTTTACATTCCCGGCAGAA
GAPDH-F	GGGAAGCTTGTCATCAATGGAA
GAPDH-R	AGAGATGATGACCCTTTTGGCTC

**注** F：正向引物；R：反向引物

10. Western blot法检测Drp1、Fis1、MFF、Pink1、Parkin蛋白表达：分别收集不同浓度代谢产物处理72 h后的处于对数生长期的KM3细胞，调整细胞密度为1×10^6^/ml，RIPA裂解混合液提取总蛋白，BCA法测定蛋白浓度。提取细胞总蛋白进行SDS-PAGE凝胶电泳，电转至PVDF膜上，无蛋白快速封闭液封闭20 min，加入稀释后的一抗（稀释比例：Drp1：1∶5 000；Fis1：1∶2 000；MFF：1∶10 000；Pink1：1∶1 000；Parkin：1∶4 000；GAPDH：1∶20 000），4 °C孵育过夜，TBST洗膜3次，加入稀释后的HRP标记的山羊抗兔二抗（稀释比例1∶5 000），室温孵育1 h，TBST洗膜5次，电化学发光法曝光显影，以GAPDH为内参，计算各组蛋白的相对表达量。

11. 补肾活血通络方化学成分分析

（1）样品溶液制备：取适量混合均匀的样品置于2 ml离心管中，加入1 ml 70％甲醇溶液和3 mm规格钢珠，用全自动样品快速研磨仪研磨3 min，取出，涡旋10 min混匀；将混匀后的样品溶液于4 °C低温条件下，以12 000 r/min（离心半径8 cm）离心10 min，上清液过0.22 µm微孔滤膜。

（2）色谱条件：采用Zorbax Eclipse C18色谱柱（2.1 mm×100 mm，1.8 µm），流动相为0.1％甲酸水溶液（A）和乙腈（B），流速为0.3 ml/min，柱温为30 °C，进样量为2 µl，梯度洗脱为0～2 min，5％B；2～7 min，30％B；7～14 min，78％B；14～20 min，95％B；20～25 min，5％B。

（3）质谱条件：电喷雾离子源（ESI），正负离子模式下扫描，正离子模式：加热器温度325 °C。鞘气流速10 L/min，辅助气流速：3 L/min，电喷雾电压：3.5 kV，毛细管温度：330 °C。HPLC-MS/MS数据采用全扫描方式采集，扫描范围为质荷比（m/z）100～1 500。

12. 网络药理学分析：将HPLC-MS/MS法鉴定所得补肾活血通络方的化学成分作为研究对象，将化合物结构导入小分子药物靶点预测在线平台（SwissTarget Prediction）数据库中进行靶点收集，删除重复靶点，即为补肾活血通络方活性成分作用靶点。在TTD、GeneCards、OMIM、DrugBank数据库中以“multiple myeloma”为搜索词获取MM疾病靶点信息，合并删除重复项得到MM疾病靶点。将补肾活血通络方与MM间的交集靶点导入DAVID数据库进行GO分析及KEGG通路富集，将结果输入微生信平台绘图。

13. 统计学处理：采用Graphpad Prism 9.0软件进行统计分析，结果以至少3次独立实验的*x*±*s*表示，采用单因素方差分析及*t*检验进行组间比较，*P*<0.05为差异有统计学意义。

## 结果

1. 补肾活血通络方代谢产物对KM3细胞增殖活性的影响：不同浓度补肾活血通络方代谢产物处理KM3细胞12、24、48、72 h后，细胞增殖均受到抑制（图1）。随着代谢产物浓度升高，KM3细胞活力显著降低，且呈浓度和时间依赖性，3％、6％、9％、12％代谢产物处理KM3细胞72 h后的增殖抑制率分别为（24.86±2.34）％、（44.89±2.06）％、（51.27±3.03）％、（61.70±2.04）％，提示补肾活血通络方代谢产物可以抑制细胞增殖活性。

**图1:**
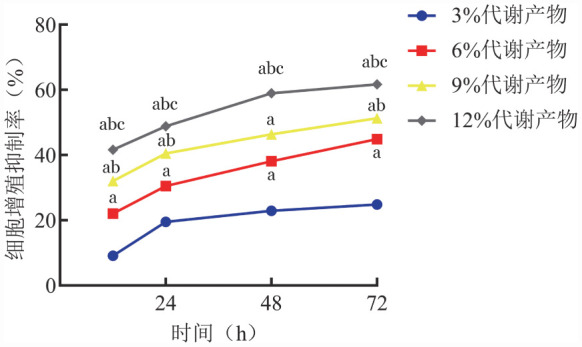
补肾活血通络方代谢产物对KM3细胞增殖活性的影响 **注** ^a^与3％代谢产物组比较，*P*<0.05；^b^与6％代谢产物组比较，*P*<0.05；^c^与9％代谢产物组比较，*P*<0.05

2. 补肾活血通络方对KM3细胞凋亡的影响：流式细胞术结果显示，与对照组比较，随着代谢产物浓度增高，KM3细胞凋亡率显著提升（图2A）。经0％、3％、6％、9％、12％代谢产物处理72 h后，细胞凋亡率分别为（6.72±0.59）％、（9.11±0.46）％、（12.98±0.47）％、（17.64±0.42）％、（20.26±0.88）％，与对照组比较，*P*值均<0.05。TUNEL染色结果显示：经不同浓度代谢产物处理72 h后细胞凋亡率分别为（4.88±1.49）％、（9.28±0.93）％、（15.22±1.14）％、（24.21±1.76）％、（32.9±2.79）％（图2B），与对照组比较，*P*值均<0.05。提示补肾活血通络方代谢产物可诱导KM3细胞凋亡，且具有浓度依赖性。

**图2:**
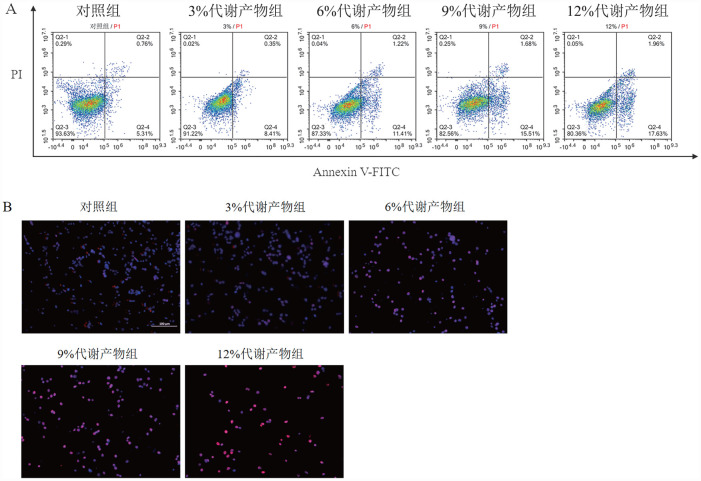
补肾活血通络方代谢产物对KM3细胞凋亡的影响 **A** 流式细胞术检测KM3细胞凋亡情况；**B** TUNEL染色法检测细胞凋亡情况

3. 补肾活血通络方对KM3细胞结构的影响：经不同浓度代谢产物干预后的5组细胞的结构存在一定差异（图3），对照组细胞线粒体结构尚可，少部分存在自噬。与对照组比较，其余4组均见线粒体损伤、线粒体分裂（红色箭头）。3％代谢产物组细胞整体无明显损伤，线粒体结构尚可；6％、9％代谢产物组细胞线粒体损伤较轻，自噬数量较少；12％代谢产物组细胞线粒体损伤相对明显，自噬数量较多。以上结果表明，补肾活血通络方能促进KM3细胞线粒体分裂、诱导细胞自噬。

**图3:**
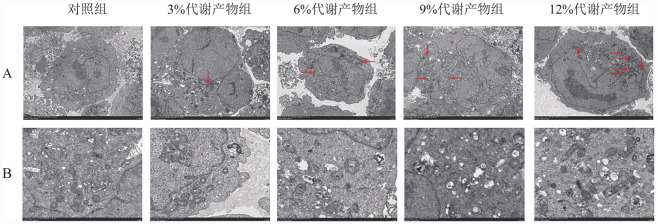
补肾活血通络方代谢产物对KM3细胞结构的影响 **A** KM3细胞内线粒体分裂情况（电镜，×2500）；**B** KM3细胞结构（电镜，×7000）

4. 代谢产物对KM3细胞株Drp1、Fis1、MFF、Pink1、Parkin mRNA和蛋白表达水平的影响：与对照组相比，不同浓度代谢产物干预后的KM3细胞中Drp1、Fis1、MFF、Pink1、Parkin的mRNA表达水平均显著升高（*P*值均<0.05）（表2）。与对照组相比，代谢产物干预后细胞中Drp1、MFF、Fis1、Pink1、Parkin蛋白表达水平均显著升高（*P*值均<0.05），且具有浓度依赖性（表3、图4）。以上结果提示，线粒体分裂和Pink1/Parkin通路介导了补肾活血通络方诱导的KM3细胞死亡。

**表2:** 不同浓度补肾活血通络方代谢产物干预后KM3细胞内各蛋白编码基因的mRNA表达水平（*x*±*s*）（实验重复3次）

组别	Drp1	Fis1	MFF	Pink1	Parkin
对照组	1.00±0.11	1.01±0.17	1.02±0.27	1.00±0.07	1.05±0.41
3％代谢产物组	3.27±0.13^a^	2.64±0.48^a^	2.35±0.11^a^	2.21±0.53^a^	2.07±0.07^a^
6％代谢产物组	7.37±0.38^ab^	3.18±0.71^a^	3.31±0.20^ab^	2.32±0.13^a^	2.36±0.33^a^
9％代谢产物组	8.32±0.09^ab^	5.06±0.81^abc^	3.78±0.37^ab^	3.29±0.40^a^	2.94±0.16^a^
12％代谢产物组	11.22±1.71^abcd^	7.05±0.61^abcd^	5.51±0.28^abcd^	6.19±0.74^abcd^	3.34±0.64^ab^

*F*值	80.033^e^	44.926^e^	122.254^e^	57.033^e^	16.316^e^

**注** ^a^与对照组比较，^b^与3％代谢产物组比较，^c^与6％代谢产物组比较，^d^与9％代谢产物组比较，*P*<0.05；^e^单因素方差分析，5组比较，*P*<0.01

**表3:** 不同浓度补肾活血通络方代谢产物干预后KM3细胞内各蛋白表达水平（*x*±*s*）（实验重复3次）

组别	Drp1/GAPDH	Fis1/GAPDH	MFF/GAPDH	Pink1/GAPDH	Parkin/GAPDH
对照组	0.58±0.14	0.31±0.01	0.39±0.01	0.66±0.02	0.33±0.03
3％代谢产物组	0.99±0.04^a^	0.48±0.02^a^	0.80±0.10^a^	0.84±0.02^a^	0.54±0.02^a^
6％代谢产物组	1.15±0.21^a^	0.54±0.08^a^	1.09±0.14^ab^	0.93±0.11^a^	0.62±0.03^a^
9％代谢产物组	1.42±0.06^ab^	0.87±0.09^abc^	1.30±0.08^ab^	1.03±0.08^ab^	0.84±0.14^abc^
12％代谢产物组	1.66±0.12^abc^	1.11±0.05^abcd^	1.46±0.13^abc^	1.14±0.03^abc^	1.07±0.06^abcd^

*F*值	30.102^e^	85.282^e^	50.054^e^	25.377^e^	47.531^e^

**注** ^a^与对照组比较，^b^与3％代谢产物组比较，^c^与6％代谢产物组比较，^d^与9％代谢产物组比较，*P*<0.05；^e^单因素方差分析，5组比较，*P*<0.01

**图4:**
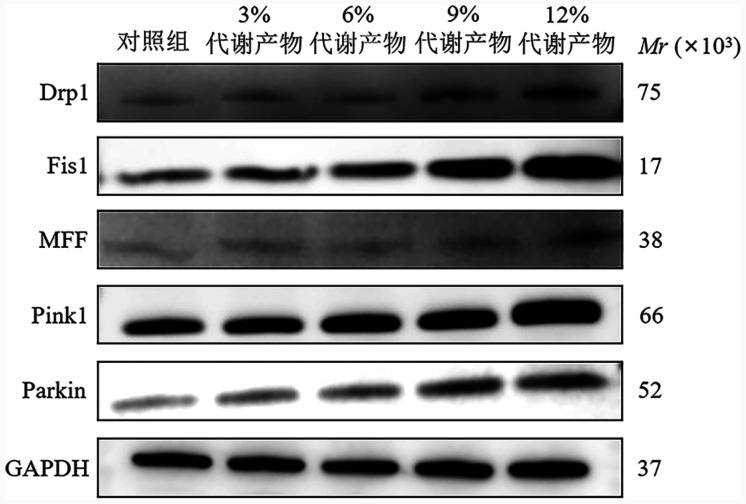
不同浓度补肾活血通络方代谢产物对KM3细胞内各蛋白表达的影响

5. 补肾活血通络方的化学成分分析：通过色谱与质谱条件对补肾活血通络方标准品溶液进行数据采集，在正、负离子两种模式下采集样品的总离子流图。共鉴定出121个化合物，包括孕烯醇酮脂类21个；脂肪酰类13个；黄酮类10个；苯及其取代衍生物类、羧酸类各9个；有机氧化合物类、小檗碱生物碱类各8个；异黄酮类6个；香豆素类5个；萘类、肉桂酸类各3个；二氢呋喃类、吗啡类、萘并呋喃类、四氢化萘类、线性1,3-二芳基丙烷类各2个；Aurone黄酮类、阿朴啡类、吡啶类、蒽类、二恶烷类、二芳基庚类、酚类、喹啉类、类固醇类、木脂素苷类、内酰胺类、嘌呤核苷类、酮酸类、异苯并呋喃类、吲哚类、有机氮化合物类各1个。

6. 补肾活血通络方“有效成分-靶点-疾病”网络的构建：共获取补肾活血通络方有效成分预测作用靶点1 043个，MM相关疾病靶点4 708个，得到474个交集靶点。将有效成分及交集靶点导入CytoScape 3.10.0软件得到“活性成分-有效靶点-疾病”网络图，Degree值前5位的化学成分为芹菜素（Apigenin）、黄藤素（Palmatine）、环香豆素（Cyclocumarol）、药根碱（Jatrorrhizine）、柚皮素（Naringenin）。

7. GO功能与KEGG通路富集分析：GO功能富集分析显示，474个交集靶点在生物学过程中主要涉及蛋白质磷酸化、凋亡过程负向调节等过程，细胞组成包括细胞核、细胞质、线粒体等，分子功能涉及组蛋白激酶活性等（图5A）。KEGG路径分析表明，药物作用与肿瘤坏死因子、核因子κB、线粒体自噬等通路相关（图5B）。以上结果证明线粒体相关通路参与了补肾活血通络方诱导KM3细胞死亡的过程，与前述实验结果一致。

**图5:**
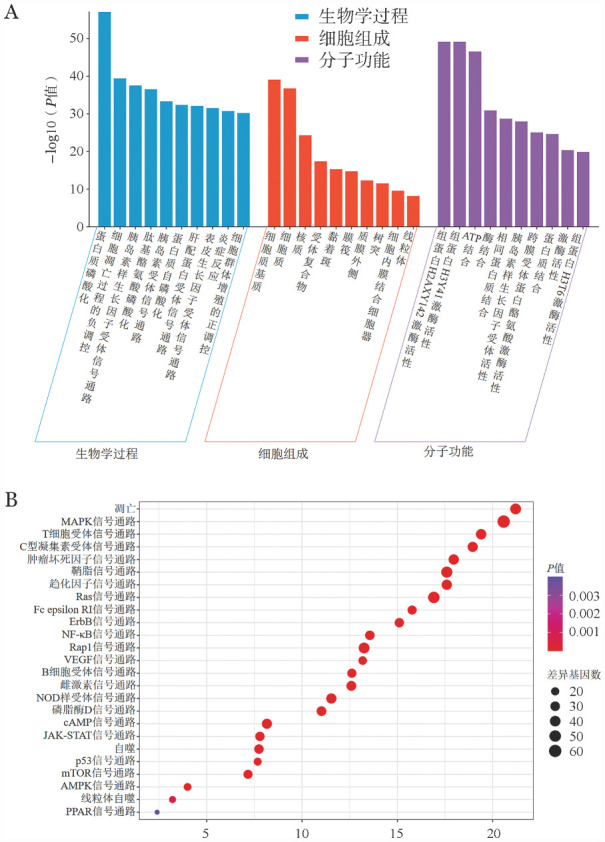
网络药理学结果图 **A** GO富集分析；**B** KEGG富集分析

## 讨论

MM是发病率排名第二的血液系统恶性肿瘤[11]，严重威胁患者健康。蛋白酶体抑制剂等治疗方法的应用很大程度上改善了患者的预后，但耐药和不良反应依旧影响患者的生活质量。补肾活血通络方为山东中医药大学附属医院经验方，前期研究证明，其联合硼替佐米诱导治疗初诊MM具有良好的效果，进一步提高了治疗总有效率，外周神经损伤等并发症发生率较低[10]。且研究证实，补肾活血通络方对MM患者的治疗具有良好的辅助作用，疗效得到广泛认可[10],[12]。

此前诸多研究表明，补肾活血通络方中的中药单体成分能够通过线粒体分裂和自噬途径影响细胞存活或抑制肿瘤细胞增殖，杜仲提取物能通过激活软骨细胞中的GLP-1R/AMPK/mTOR信号通路诱导细胞自噬[13]，并在体壁肌肉中刺激线粒体分裂，减轻与细胞衰老相关的线粒体损伤[14]。补骨酯素能通过Wnt/β-catenin信号通路激活DRP1介导的线粒体分裂和内质网应激相关的细胞凋亡[15]。黄精凝集素能通过线粒体介导的ROS-p38-p53通路诱导细胞凋亡与自噬[16]。山茱萸苷能下调由自噬相关通路调控的NOX4的表达进而抑制细胞凋亡[17]。

线粒体分裂是线粒体动力学的重要组成部分，在分裂过程中会清除受损成分并完成质量控制[7]，线粒体分裂通过与线粒体融合的协同作用保持线粒体网络的动态平衡[18]。Drp1是一种胞质蛋白，从细胞质被募集到线粒体外膜后被磷酸化激活，促进线粒体分裂[19]。Fis1和MFF是线粒体外膜上接受外来信号并启动线粒体分裂的主要受体蛋白，在外界因素的干预下能够被活化，促进线粒体外膜和内膜收缩，完成线粒体分裂过程[20]。既往研究表明，诱导线粒体分裂能抑制MM细胞活性[5]–[6]。自噬是细胞内重要的分解和回收机制，参与MM细胞的恶性增殖、诱导耐药等过程，与MM的发生发展关系密切[21]。Pink1和Parkin是两种促自噬蛋白，二者协同识别损伤的线粒体，Pink1可在识别损伤线粒体时稳定聚集于外膜表面，发挥分子感受器作用，Parkin泛素化线粒体外膜蛋白引发线粒体自噬[22]。在肿瘤疾病进展过程中，自噬显示出促进癌症进展或诱导肿瘤细胞凋亡的双向作用，相关研究表明，中药有效成分能够抑制MM细胞的体外增殖活力，其作用机制与激活细胞自噬性凋亡有关[23]。

本研究显示，补肾活血通络方代谢产物能抑制KM3细胞活力，并呈时间和浓度依赖性。流式细胞术和TUNEL染色结果显示，与对照组比较，补肾活血通络方可促进KM3细胞凋亡，其中12％代谢产物组效果最显著，其作用机制可能与激活自噬活性、诱导细胞能量耗竭、促进MM细胞凋亡相关。通过透射电镜观察MM细胞结构，显示补肾活血通络方会导致KM3细胞线粒体损伤，与对照组比较，各实验组均见线粒体分裂，其中12％代谢产物组细胞线粒体损伤最明显，自噬数量较多，自噬溶酶体大量存在，提示补肾活血通络方对KM3细胞的抑制作用可能通过影响线粒体分裂和自噬进行。荧光定量PCR和Western blot结果显示，与对照组比较，各实验组中Drp1、Fis1、MFF、Pink1、Parkin的mRNA和蛋白表达均显著升高（*P*值均<0.05），且呈浓度依赖性。提示补肾活血通络方对KM3细胞的作用很可能通过增加线粒体动力学分裂、诱导细胞自噬完成。通过网络药理学进一步验证线粒体相关通路在补肾活血通络方治疗MM中的作用，KEGG分析表明，补肾活血通络方的药物作用与线粒体自噬通路有关，侧面印证了线粒体相关通路参与了补肾活血通络方对MM的干预。此外，本研究仍存在一定局限性，本研究为细胞体外实验，补肾活血通络方在MM动物模型中的作用效果和机制尚不明确，仍需进一步研究。

综上所述，本研究展示了补肾活血通络方代谢产物对MM KM3细胞的增殖抑制作用并进行了网络药理学验证，其作用机制可能与上调Drp1、Fis1、MFF、Pink1、Parkin表达，促进线粒体分裂，诱导细胞发生自噬有关，抑制增殖和诱导自噬作用呈时间和剂量依赖性，显示了补肾活血通络方的抗肿瘤活性及其辅助治疗MM的应用前景，为中西医结合治疗MM提供了新思路。
